# The U. S. Army Laboratory at Philadelphia

**Published:** 1863-11

**Authors:** 


### THE UNITED STATES ARMY LABORATORY AT PHILADELPHIA.

Since our last notice of this enterprise of Surgeon-General Hammond's, we have twice visited the laboratory, where Dr. A. K. Smith, U. S. Army, the Director, and Prof. Maisch, the Chemist, politely showed us the several departments at present in operation. The Laboratory buildings are those formerly occupied by Crew, Rogers & Crew, for their chemical works, at Sixth and Oxford streets. The main building has three stories, with a large one-story building attached, and several detached structures for special purposes. All the heating in the main building is effected by steam, except such as is performed by gas burners. A twenty five horse power engine, with appropriate boilers, is erected in a position central to the laboratory operating rooms, and yet separate. Immediately above the boilers, and deriving its heat from them, is the drying-room, which opens by a door into the mill or powdering room. In the latter there are at present two pairs of chasers, and one Bogardus' mill. Two more pairs of chasers are to be erected in a short time. Mr. Maisch informs us that he has succeeded in getting his bolting machine to operate very successfully. In this room is also the machine for making the preparations of free metallic mercury, as blue pills, mercurial ointment, etc., by shaking, as described by Dr. Squibb, except that the plan of the machine is more simple. In this room, all the fine powders, as ipecac, rhubarb, jalap, etc., are prepared, and sent up stairs to be bottled, whilst the chief occupation of the mill is in preparing drugs for precolation. Proceeding eastward from the mill room, the visitor enters the general operating room for Pharmaceutical and Chemical processes; commencing with the percolators, which are adjacent to the mill room, the processes become more chemical towards the further end. The visitor can here witness the concentration of liquids on water baths, and in stills for fluid and solid extracts, preparations of morphia, and for the crystallization of salts. The preparation of the officinal solutions of ammonia is conducted here also, but apart from the other processes.

The large percolators are constituted of wood, lined with tinned copper, varying in capacity from 260 gallons to 150 gallons. Besides these, vessels of smaller size, constructed of tinned iron, are in use for lesser operations. 280 pounds of colocynth, and 600 pounds of valerian are percolated at one operation. These wooden percolators are arranged on a stage, on a level with, and connected with the mill room, so as to be easily charged. Each percolator has a manhole in front near the bottom, closed by clamps and screws, through which the exhausted material is extracted after each operation. Hanging in front of each percolator is a black-board, on which is written the leading facts of each operation as they are developed, such as name and quantity of material, menstruum, and percolate, with remarks when necessary. Along the eastern end, a range of jacketted steam evaporators are in operation, and jacketted stills. In a detatched brick building, on the north side of the lot, is the room for furnace operations, including the preparation of oil of wine, which will be made in eight-gallon retorts, on sand baths. Here the oxidation and solution of metals, and numerous other operations involving direct heat, will be conducted. In the centre of the area, a building is being constructed specially for the manufacture and bottling of ether, sweet spirits of nitre, and chloroform, with a subterranean store room. Steam heat only will be used, and no light or fire of any kind allowed in the building. The apparatus for ether will be that of Dr. Squibb, described before in this journal. By thus isolating these articles, much of the usual danger of fire will be avoided. Ample space remains in the yard for extending the buildings if required.

Returning to the main building, we find the storekeeper's room next to the mill room on the first floor, and north of this, other rooms, among which are the office and Mr. Maisch's private analytical laboratory, neatly fitted up with apparatus needed in the examination of drugs and chemicals previous to their purchase, when required. On the second floor north is the sewing machine room, in which twelve girls and a cutter, operating ten sewing machines, make one thousand linen sheets daily, and pillow cases, towels, and other items required in the army hospitals. On the opposite end of the building is the filling room, where all powders, salts, pills, and other dry substances are put up in bottles for the medicine chests; and in a similar room directly above this all the various fluid extracts, tinctures, and other liquids are bottled and labelled, each kind put up by itself on shelves for temporary storage, above the counter. The work in these two rooms occupy twelve girls, besides six others engaged in washing the bottles. In the pill room, four girls are engaged in making pills. At present the common pill machine only is employed, the composition and formation of the mass is superintended by a Graduate of Pharmacy. The pills made are pil. opii, pil cathart., comp. and pil. hydrarg., U. S. P.; and pil. camphoræ, et opii, pil. colcynth comp. et. ipecac, and pil. quiniæ sulph. *aa* 3 grs.

It should be understood that the medical supply-table for the army is by no means so comprehensive as the Pharmacopœia, and consequently the scope of operations is confined chiefly to those preparations on the list. It is intended to make Ceratum Simp., Cantharidis, and Resinæ, and, as soon as arrangements can be made, to spread adhesive plaster and isinglass plaster for the entire army. Morphia will also be made to an extent adequate to the wants of the whole army. It has been determined to manufacture sulphate of quinia; and as soon as the *bark* arrives this will be commenced, and the experiment of its economy made. About two hundred serons of Cinchona have been purchased.

The basement of the main building is paved with brick throughout, and is used for storing and bottling liquors, and fixed oils. Three girls attend to the bottling of liquors. The medical store wagons and panniers are filled at the laboratory, but made elsewhere. The bottles used are marked in the moulding, "U. S. A. Hosp. Dept.;" and are furnished from Pittsburgh. Each bottle of any size is enclosed in a square pasteboard box surrounded with sawdust or rice husks, and these closely packed in wooden boxes appropriately marked, and then conveyed to the storehouse at Sixth and Master streets.

All drugs are purchased on the requisition of the Director, Dr. Smith, by an order from the medical purveyor (Dr. Robert Murray, U. S. A.) to a drug broker, it being clearly understood that all purchases are subject to the inspection and analysis of Mr. Maisch.

Such is a hasty view of this new enterprise. So far, we are informed, on many leading articles great economy has attended the experiments, and all has been well done. In the sewing machine department, since operations commenced, Dr. Smith says that they have paid for the machines, and saved the Government $1200 besides! Of course it will take a longer period to determine the actual facts of the case, but there can be but little doubt of the expediency of the measure, whilst the necessity for large supplies exists, and under the care of such earnest workers as Dr. A. K. Smith and Prof. Maisch it will receive a fair trial.—*Journal of Pharmacy.*

